# Transpapillary stenting by the rendezvous technique using a novel device delivery guide sheath via percutaneous transhepatic biliary drainage route for hilar biliary obstruction

**DOI:** 10.1055/a-2559-4266

**Published:** 2025-03-25

**Authors:** Masaki Miyazawa, Kazuki Nagai, Masaki Nishitani, Tomoyuki Hayashi, Shinya Yamada, Hajime Takatori, Taro Yamashita

**Affiliations:** 1 88335 Department of Gastroenterology, Kanazawa University Hospital, Kanazawa, Japan

The rendezvous technique via percutaneous transhepatic biliary drainage (PTBD) or endoscopic ultrasonography-guided biliary drainage is a useful salvage procedure when transpapillary stenting is unsuccessful 1 2 . The success of this procedure requires that the guidewire on the fistula side break through the bile duct stricture, which can sometimes be difficult. Herein, we report successful transpapillary stenting by the rendezvous technique using a novel device delivery guide sheath via the PTBD route to treat hilar biliary obstruction (HBO).

A 47-year-old woman who had suffered biliary injury during cholecystectomy for cholecystitis presented with acute cholangitis due to HBO ( Fig. 1 ). Although a plastic stent was placed in the right hepatic duct (RHD), it was impossible to approach the left hepatic duct (LHD), so PTBD was performed in the LHD. We then attempted transpapillary stenting using the rendezvous technique via the PTBD route ( Video 1 ).

**Fig. 1:**
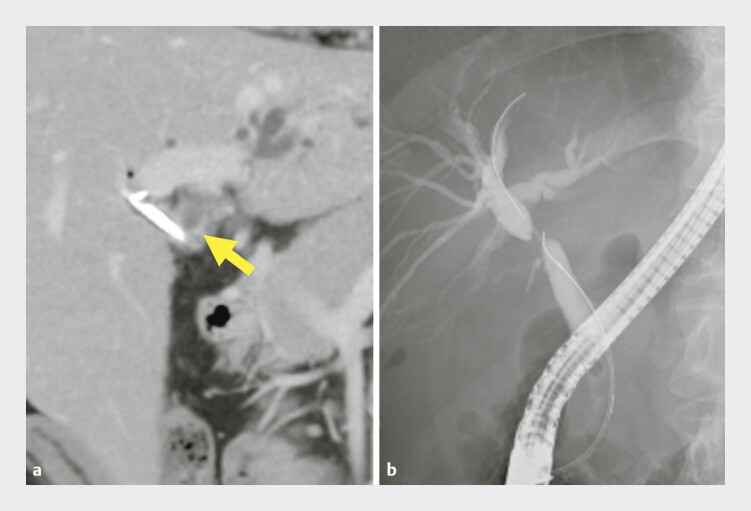
**a** Abdominal computed tomography revealed an undrained left intrahepatic bile duct (arrow). **b** Cholangiography showed Bismuth-Corlette classification type II hilar biliary obstruction.

Successful transpapillary stenting by the rendezvous technique using a novel device delivery guide sheath via the percutaneous transhepatic biliary drainage route for hilar biliary obstruction.Video 1

A guidewire via the PTBD route was not inserted into the common bile duct (CBD) due to HBO and was instead inserted only into the RHD ( Fig. 2 **a** ). Therefore, the rendezvous technique was attempted by grasping the guidewire within the RHD via a transpapillary approach and pulling it into the CBD. A novel device delivery guide sheath (Endosheather; Piolax Medical Devices, Kanagawa, Japan) ( Fig. 2 **b** ) was used to break through the HBO. The inner catheter was then removed and a 1.8-mm-diameter pediatric biopsy forceps (Radial Jaw 4P; Boston Scientific, Massachusetts, USA) inserted into the RHD through the outer sheath. The tip of the guidewire via the PTBD route was grasped with the biopsy forceps ( Fig. 2 **c** ), and slowly pulled into the CBD together with the guide sheath, successfully dropping the guidewire into the CBD ( Fig. 2 **d** ). Finally, a plastic stent was placed in both the LHD and the RHD via a transpapillary approach using the rendezvous technique ( Fig. 2 **e** ).

**Fig. 2:**
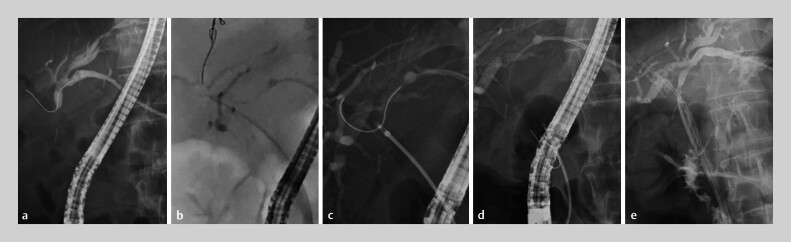
**a** A guidewire via the PTBD route was not inserted into the common bile duct due to the hilar stricture and was instead inserted only into the right hepatic duct. **b** The hilar stricture was broken through using a novel device delivery guide sheath (Endosheather; Piolax Medical Device, Kanagawa, Japan). **c** The tip of the guidewire via the PTBD route was grasped with a 1.8-mm-diameter pediatric biopsy forceps inserted into the right hepatic duct through the outer sheath. **d** The biopsy forceps was slowly pulled into the common bile duct together with the guide sheath, successfully dropping the guidewire into the common bile duct. **e** A plastic stent was placed in the left and right hepatic ducts via a transpapillary approach using the rendezvous technique.

We believe that transpapillary stenting by the rendezvous technique using a novel device delivery guide sheath is effective for the treatment of refractory bile duct strictures.

Endoscopy_UCTN_Code_TTT_1AR_2AG

## Notes

Endoscopy E-Videos https://eref.thieme.de/e-videos
*E-Videos* is an open access online section of the journal *Endoscopy* , reporting on interesting cases and new techniques in gastroenterological endoscopy. All papers include a high-quality video and are published with a Creative Commons CC-BY license. Endoscopy E-Videos qualify for HINARI discounts and waivers and eligibility is automatically checked during the submission process. We grant 100% waivers to articles whose corresponding authors are based in Group A countries and 50% waivers to those who are based in Group B countries as classified by Research4Life (see: https://www.research4life.org/access/eligibility/ ). This section has its own submission website at https://mc.manuscriptcentral.com/e-videos .
